# Comparative analysis of the complete plastid genomes in *Prunus* subgenus *Cerasus* (Rosaceae): Molecular structures and phylogenetic relationships

**DOI:** 10.1371/journal.pone.0266535

**Published:** 2022-04-06

**Authors:** Meng Li, Yan-Feng Song, Steven P. Sylvester, Steven P. Sylvester, Xian-Rong Wang

**Affiliations:** Co-Innovation Center for Sustainable Forestry in Southern China, College of Biology and the Environment, Nanjing Forestry University, Nanjing, Jiangsu, China; National Agri-Food Biotechnology Institute (NABI) Mohali, INDIA

## Abstract

*Prunus* subgenus *Cerasus* (cherry) is an economically important group that distributed in temperate regions of the northern hemisphere. However, shared interspecific morphological traits and variability across taxa of *Cerasus* are among the impediments to taxonomic efforts to correctly delimit taxa. This is further complicated by a lack of genetic information on these taxa, with no focused genomic or phylogenetic studies being done on *Cerasus*. In this study, we conducted comparative analysis on the complete plastid genomes (plastomes) of 20 *Cerasus* species to gain a greater understanding of the attributes of the plastome of these taxa while helping resolve their phylogenetic placement in *Prunus sensu lato* and interspecific relationships within the subgenus. Our results displayed that (1) the plastomes of the 20 *Cerasus* species studied exhibited a typical quadripartite structure with conversed genome arrangement, structure, and moderate divergence. (2) The average size of complete plastomes for the *Cerasus* taxa studied was 157,861 bp, ranging from 157,458 to 158,024 bp. A total of 134 genes were annotated, including 86 protein-coding genes, 40 tRNAs, and 8 rRNAs across all species. In simple sequence repeat analysis, we found *Cerasus* had a comparable number of dispersed and tandem repeats to those identified in other angiosperm taxa, with only *P*. *pseudocerasus* found to contain trinucleotide repeats. Nucleotide diversity analysis revealed that the *trnG-GCC* gene and *rpl32*-*trnL* region had the highest Pi value showing potential as phylogenetic markers. (3) Two phylogenetic trees of the plastomes verified the monophyletic relationship of *Cerasus* and provided a more resolved species-level phylogeny. Our study provides detailed plastome information for exploring the phylogeny of subg. *Cerasus* taxa. We identified various types of repeats and nucleotide diversity hotspots, which can be a reference for species identification and reconstruction of phylogenetic relationships.

## Introduction

*Prunus* L. subg. *Cerasus* (Mill.) A. Gray contains approximately 150 species that mainly distributed in temperate and subtropical regions of the northern hemisphere [1–5]. Subg. *Cerasus* provides various edible cherries and ornamentals of economic value, and has great potential for development and application, the research on this economically important group is becoming more and more extensive. Through long-term hybridization and domestication of subg. *Cerasus* species, a large number of economically significant species, such as sweet cherry (*P*. *avium*), Chinese cherry (*P*. *pseudocerasus*), and variety of ornamental specie has been widely planted. In recent years, many new cultivars have been developed, indicating that the subg. *Cerasus* has great potential for development and utilization. However, interspecific hybridizations have complicated the taxonomy of this subgenus [6], making it necessary to investigate the phylogenetic relationships of wild subg. *Cerasus* species resources. Although some studies have clarified the deep phylogenetic relationships and diversification history of Rosaceae and *Prunus s*.*l*. [7–10], only one phylogenetic study has included 13 taxa from subg. *Cerasus* and was based on a minimal number of molecular markers [8].

The annotation and bioinformatics analysis of plastid genome play an important role in the classification and evolution of plant species, due to its unique genetic characteristics of maternal inheritance. A typical plastid genome (plastome) consists of a pair of inverted repeats (IR) regions that is separated by a large single copy (LSC) region and a small single copy (SSC) region, with standard plastome sizes ranging between 120 and 170 kb in length [11]. Compared with the nuclear genome, the plastome in angiosperms is a circular DNA molecule which has a highly conserved genomic structure with a small size, single-parental inheritance, and low nucleotide substitution rate. Based on these advantages, the plastome is used in diverse studies focused on species identification, population genetic analyses, and phylogenetic analyses, with phylogenetic studies using the complete plastome undertaken in many angiosperm groups [12–16].

In this study, we compared and analyzed the published complete plastomes of 20 subg. *Cerasus* species from GenBank, aiming to reveal the complete structure of plastomes and hotspot regions among these 20 species while clarifying their phylogenetic placement. This information will be valuable for further evolutionary studies on subg. *Cerasus* and Rosaceae in general.

## Materials and methods

### Sampling, DNA isolation and sequencing

There are no specific permits required for obtaining the healthy and fresh leaves of *P*. *jamasakura* and *P*. *discoidea*, since they are not endangered or protected species and were collected from the fields that are not privately owned or protected. We collected fresh young leaf samples for *P*. *jamasakura* and *P*. *discoidea* from Katano, Japan (34°45’53.7"N, 135°42’10.5"E) and Huangshan, China (30°4’14.08"N 118°5’25.54"E. We prepared the voucher specimen for two samples used and deposited them in Nanjing Forestry University (voucher numbers: NF161093652, NF161093753). The leaf samples were quickly dried with silica gel in a zip lock plastic bag upon the sampling and stored at room temperature until further see.

Total Genomic DNA were extracted from each of the two subg. *Cerasus* plants using a DNeasy Plant Mini Kit (Qiagen Co., Hilden, Germany) following the manufacturer’s protocol. The extracted DNA were quantified in NanoDrop ND1000 (Thermo Fisher Scientific, Massachusetts, USA; quality cutoff, OD 260/280 ratio between 1.7–1.9) and visualized in a 1% agarose-gel electrophoresis for the quality check. Illumina paired-end (PE) libraries (read length: 2 × 125 bp) with insert sizes of 270 to 700 bp for each of the two *Cerasus* species were constructed and sequenced on MiSeq platform (Illumina Inc., San Diego, CA) by Nanjing Genepioneer Biotechnologies Inc. (Nanjing, China). We removed poor quality reads (PHRED score of < 20) using the quality trim function implemented in CLC Assembly Cell package v. 4.2.1 (CLC Inc., Denmark).

### Genome assembly and annotation

We employed the low-coverage whole-genome sequence (dnaLCW) method [17] to assemble the complete CP genomes using both CLC de novo assembler in CLC Assembly Cell package and SOAPdenovo (SOAP package v. 1.12) with default parameters. Gaps were filled by the Gapcloser function in the SOAP package. To improve the CP genome assembly, we also conducted reference-based genome assembly using the CP genome sequences of *P*. *cerasoides* (GenBank accession: MF621234). The contigs obtained from the primary de novo assemblies were aligned to the reference CP genome, then the aligned contigs were assembled to each chloroplast genome in Geneious v7 (http://www.geneious.com). We annotated the CP genomes assembled using the online tool, DOGMA (Dual Organellar GenoMe Annotator) [18] with a few adjustments for start and stop codons. Protein-coding genes were defined based on the plastid-bacterial genetic code. We also scanned all tRNAs with tRNAscan-SE [19] using the default settings to confirm the tRNA boundaries identified by DOGMA. Since this study adopted the concept of *Prunus s*.*l*., genus name *Prunus* was used to represent the subgenus name *Cerasus*. The circular plastome maps were visualized using OGDRAW v 1.3.1. The other annotated plastome sequences of 18 subg. *Cerasus* species we studied were all downloaded from GenBank and the accession numbers listed in Table 1.

**Table 1:** Sample information and summary of plastome characteristics for the 20 subg. *Cerasus* species studied.

Species	Reference Sequence	Total Length (bp)	LSC length (bp) (GC content)	SSC length (bp) (GC content)	IR length (bp) (GC content)	GC content	Total number of genes
*Prunus avium*	NC044701	157886	85990(34.54%)	19080(30.24%)	26408(42.52%)	36.69%	131
*P*. *campanulata*	NC044123	157938	85947(34.59%)	19119(30.21%)	26436(42.52%)	36.72%	128
*P*. *cerasoides*	NC035891	157685	85792(34.59%)	19061(30.26%)	26416(42.51%)	36.71%	129
*P*. *conradinae*	MT374065	158019	85910(34.60%)	19247(30.06%)	26431(42.53%)	36.70%	130
*P*. *discoidea*	MN158647	158024	85953(34.59%)	19133(30.20%)	26469(42.50%)	36.70%	130
*P*. *emarginata*	MN389436	157458	85567(34.54%)	19121(30.12%)	26385(42.53%)	36.68%	129
*P*. *itosakura*	MN695296	157813	85931(34.62%)	19120(30.19%)	26381(42.56%)	36.73%	129
*P*. *jamasakura*	MN652612	157905	85910(34.59%)	19123(30.19%)	26436(42.52%)	36.71%	129
*P*. *kumanoensis*	MN245147	157898	85926(34.59%)	19070(30.26%)	26451(42.52%)	36.73%	129
*P*. *leveilleana*	MN913372	157935	85940(34.58%)	19121(30.22%)	26437(42.52%)	36.71%	129
*P*. *matuurae*	NC045230	157928	85904(34.59%)	19104(30.23%)	26460(42.50%)	36.71%	129
*P*. *maximowiczii*	NC026981	157852	85847(34.62%)	19133(30.12%)	26436(42.56%)	36.72%	129
*P*. *pensylvanica*	MN427872	157953	86030(34.55%)	19135(30.15%)	26394(42.52%)	36.68%	129
*P*. *pseudocerasus*	KX255667	157834	85954(34.64%)	19084(30.36%)	26398(42.46%)	36.70%	131
*P*. *rufa*	NC048528	157723	85860(34.60%)	19081(30.23%)	26391(42.55%)	36.73%	129
*P*. *spontanea*	KP760073	157882	85968(34.60%)	19120(30.22%)	26397(42.53%)	36.72%	129
*P*. *speciosa*	MH998233	157916	85927(34.59%)	19123(30.19%)	26433(42.53%)	36.71%	129
*P*. *subhirtella*	KP760075	157833	85951(34.59%)	19120(30.20%)	26381(42.56%)	36.73%	129
*P*. *takesimensis*	NC039379	157948	85959(34.59%)	19117(30.12%)	26436(42.53%)	36.71%	129
*P*. *yedoensis*	NC026980	157792	85914(34.64%)	19120(30.26%)	26379(42.49%)	36.74%	130

### Comparative genome analysis

The IRSCOPE (https://irscope.shinyapps.io/irapp/) was chosen to compare the boundaries between single copy regions (LSC and SSC) and inverted repeat (IR) regions among the 20 subg. *Cerasus* plastome sequences. The mVISTA (http://genome.lbl.gov/vista/mvista/submit.shtml) visualized the differences between the complete plastid sequences of 20 subg. *Cerasus* species in Shuffle-LAGAN mode with the annotated complete plastome of *P*. *avium* as a reference. To analyze nucleotide diversity (Pi), we applied the window size of 600bp with a 200bp step size and we extracted the shared 93 genes of 20 species in subg. *Cerasus* after alignment. The Pi value among the 20 subg. *Cerasus* species was calculated using the DnaSP v6 [20], which was utilized to determine the average number of nucleotide differences between all taxa.

### Repeat sequences and SSR analysis

REPuter [21] was selected to investigate the tandem repeat sequences and the size of repeat sequences, which included four types of repeats in the plastomes of the 20 subg. *Cerasus* species. For REPuter analysis, we set the parameters with hamming distance of 3 bp and minimal repeat size of 30 bp. We used SSR software MicroSAtellite (MISA) (http://pgrc.ipk-gatersleben.de/misa/) to identify SSR sequences and tandem repeats of 1–6 nucleotides were considered microsatellites. The minimum numbers of repeats were set to 10, 5, 4, 3, 3, and 3 for mono-, di-, tri-, tetra-, penta-, and hexa-nucleotides, respectively.

We also analyzed codon usage to examine the distribution of codon usage using CodonW v1.4.2 (http://codonw.sourceforge.net/) with RSCU ratio for all protein-coding genes.

### Phylogenetic analysis

We selected the 20 previously sequenced plastomes from subg. *Cerasus* in GenBank (Table 1), and combined these with 29 published plastomes of Rosaceae and four other angiosperm species that were set as an outgroup for the phylogeny. The plastomes of Rosaceae were conserved in gene construction, so the alignment was straightforward. We utilized different databases, including complete plastome and CDS regions to construct the phylogenetic tree. Before building the phylogenetic tree with plastomes, 53 sequences were aligned with MAFFT v7.467 [22] and manually adjusted in BioEdit [23], and the phylogenetic analyses were performed by Maximum likelihood (ML) and Bayesian inference (BI) methods, respectively. Maximum likelihood (ML) analyses were performed using IQ-TREE v2.1.1 [24] with 1000 bootstrap replications. MrBayes v3.2.7 [25] was used to conduct Bayesian inference (BI) analyses. The Markov chain Monte Carlo (MCMC) algorithm was run for two million generations with trees sampled every 500 generations. The first 25% of generations were discarded as burn-in. A 50% majority-rule consensus tree was constructed from the remaining trees to estimate posterior probabilities (PPs). To determine the best fitting substitution model, the phylogenetic trees were visualized using Figtree v1.4 (http://tree.bio.ed.ac.uk/software/figtree/) [26].

## Results

### Plastome structure

**Fig 1:**
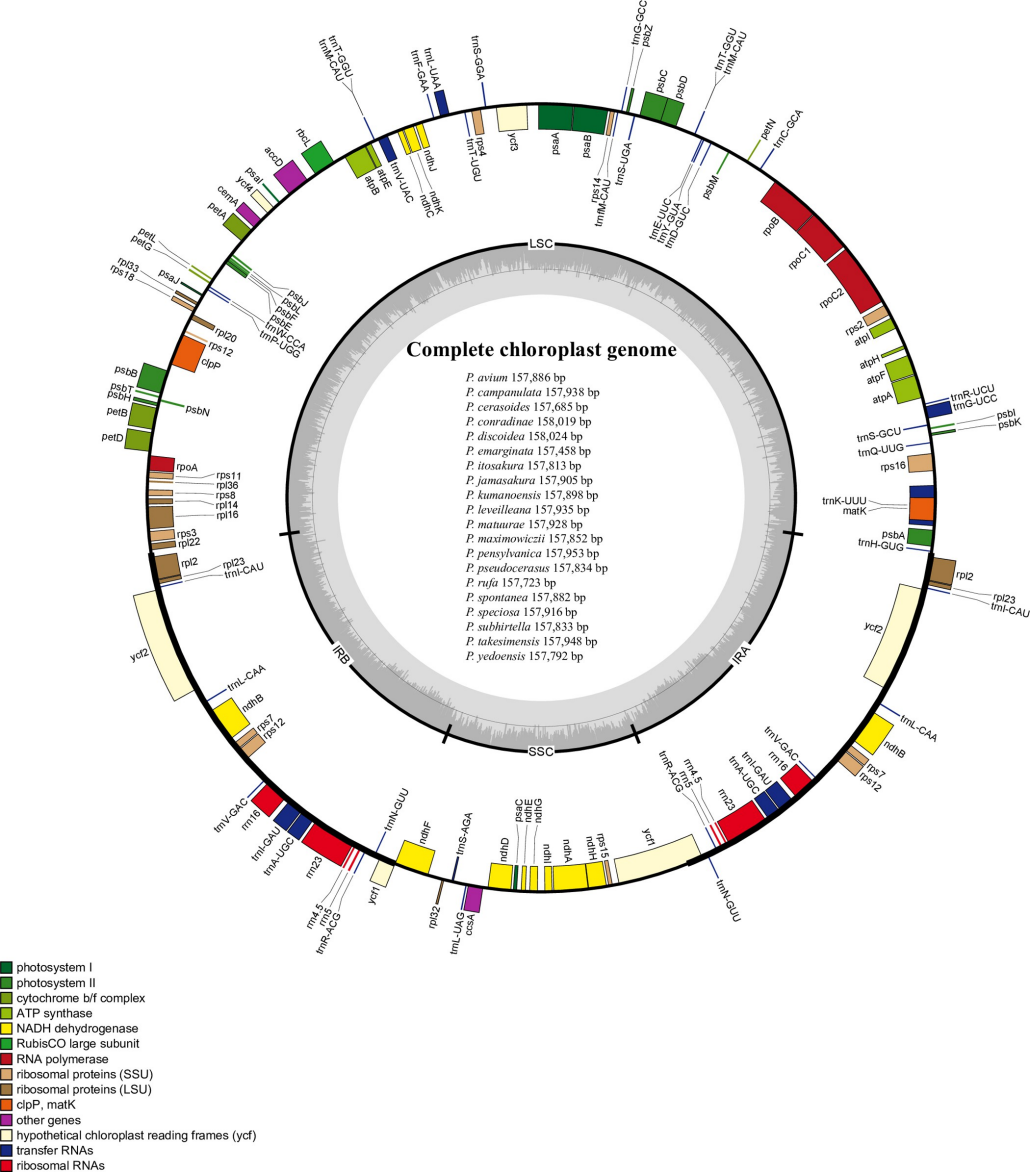
Plastid map of 20 subg. *Cerasus* species. The colored boxes represent conserved plastid genes. Genes shown inside the circle are transcribed clockwise, whereas genes outside the circle are transcribed counter-clockwise. Genes are color-coded to indicate functional groups. The dark gray area in the inner circle corresponds to GC content while the light gray corresponds to the AT content of the genome. The small (SSC) and large (LSC) single copy regions and inverted repeat (IRa and IRb) regions are noted in the inner circle.

The plastomes of the subg. *Cerasus* taxa studied have the typical quadripartite structure comprising long single copy (LSC), inverted repeat (IR) and small single copy (SSC) regions (Fig 1). The average length of complete plastomes among these subg. *Cerasus* species is 157,861bp, ranging from 157,458 to 158,024 bp in length (Fig 1 and Table 1), with IRs of 26,379–26,469 bp, LSCs 85567–86,030 bp and SSCs 19,061–19,247 bp. The entire GC content of these plastome sequences is 36.68–36.74%, and the GC contents of the LSC, SSC, and IR regions are 34.54–34.64%, 30.06–30.36%, and 42.46–42.56%, respectively. The eight rRNA genes are distributed in the IR region, resulting in the highest GC content in this region.

### Plastome annotation

The subg. *Cerasus* plastomes contained 134 genes, which consisted of 78 protein coding genes, 31 tRNA- and 4 rRNA-coding genes (Table 2). There was a single functional protein encoding gene (*rps19*) deletion found in *P*. *avium* resulting in 77 protein coding genes for this species, with *rps19* repeated twice in *P*. *pseudocerasus* (Lindl.) G. Don and *P*. *yedoensis* (Mats.) Yü et Li. The *ycf1* gene also repeats twice in *P*. *conradinae*, *P*. *discoidea*, *P*. *pseudocerasus* and *P*. *yedoensis*. Compared with tRNAs in other subg. *Cerasus* species, *P*. *campanulata* and *P*. *yedoensis* lacked the *trnfM-*CAU gene. *P*. *avium* encodes the most abundant tRNA of all subg *Cerasus* species studied. And this species contains the unique trnS-AGA gene. At the same time, *trnM*-CAU & *trnT*-GGU are repeated twice in *P*. *avium*. Ten protein coding genes (*ndhB*, *ndhA*, *petB*, *petD*, *rpl2*, *rpl16*, *rps12*, *rps19*, *rps16*, *rpoC1*) contained one intron, two genes (*clpP*, *ycf3*) contained two introns (Table 3).

**Table 2:** List of genes within plastomes of the 20 subg. *Cerasus* species studied.

Category	Group of gene	Name of gene
Photosynthetic	Photosystem Ⅰ	*psaA*, *psaB*, *psaC*, *psaI*, *psaJ*
	Photosystem Ⅱ	*psbA*, *psbB*, *psbC*, *psbD*, *psbE*, *psbF*, *psbH*, *psbI*, *psbJ*, *psbK*, *psbL*, *psbM*, *psbN*, *psbT*, *psbZ*
	NADH dehydrogenase	*ndhA*^a^, *ndhB*(×2)^a^, *ndhC*, *ndhD*, *ndhE*, *ndhF*, *ndhG*, *ndhH*, *ndhI*, *ndhJ*, *ndhK*
	Cytochrome b6/f complex	*petN*, *petA*, *petL*, *petG*, *petB*^a^, *petD*^a^
	ATP synthase	*atpA*, *atpB*, *atpE*, *atpF*^a^, *atpH*, *atpI*
	Rubisco large subunit	*rbcL*
Transcription & Translation	Ribosomal protein, LSU	*rpl2*(×2)^a^, *rpl14*, *rpl16*^a^, *rpl20*, *rpl22*, *rpl23*(×2), *rpl32*, *rpl33*, *rpl36*
	Ribosomal protein, SSU	*rps2*, *rps3*, *rps4*, *rps7*(×2), *rps8*, *rps11*, *rps12*(×2)^a^, *rps14*, *rps15*, *rps16*^a^, *rps18*, *rps19*(×2)
	RNA polymerase	*rpoA*, *rpoB*, *rpoC1*^a^, *rpoC2*
	Ribosomal RNAs	*rrn23*(×2), *rrn16*(×2), *rrn5*(×2), *rrn4*.*5*(×2)
	Transfer RNAs	*trnA-UGC*(×2), *trnC-GCA*, *trnD-GUC*, *trnE-UUC*, *trnF-GAA*, *trnfM-CAU*, *trnG-GCC*, *trnG-UCC*, *trnH-GUG*, *trnI-CAU*(×2), *trnI-GAU*(×2), *trnK-UUU*, *trnL-CAA*(×2), *trnL-UAA*, *trnL-UAG*, *trnM-CAU*(×2), *trnN-GUU*(×2), *trnP-UGG*, *trnQ-UUG*, *trnR-ACG*(×2), *trnR-UCU*, *trnS-AGA*, *trnS-GCU*, *trnS-GGA*, *trnS-UGA*, *trnT-GGU*(×2), *trnT-UGU*, *trnV-GAC*(×2), *trnV-UAC*, *trnW-CCA*, *trnY-GUA*
Biosynthesis	Maturase	*matK*
	Protease	*clpP* ^b^
	Envelope membrane protein	*cemA*
	Acetyl-CoA carboxylase	*accD*
	c-type cytochrome synthesis gene	*ccsA*
Unknown function	Conserved hypothetical plastid Reading Frames	*ycf1*(×2), *ycf2*(×2), *ycf3*^b^, *ycf4*

×2 refers to genes duplicated in the IR regions; Genes marked with asterisks are with single (^a^) or double (^b^) introns.

**Table 3:** Information on 10 intron-containing genes in the plastome of the 20 subg. *Cerasus* species studied.

Gene	Location	Start	End	ExonⅠ(bp)	IntronⅠ(bp)	ExonⅡ(bp)	IntronⅡ(bp)	ExonⅢ(bp)
*rps12*	IRa	99918	100711	26	536	233		
*rps16*	LSC	5217	6349	30	863	40		
*atpF*	LSC	11606	12907	410	747	145		
*rpoC1*	LSC	20998	23809	1617	760	435		
*ycf3*	LSC	43927	45919	153	777	228	709	126
*rps12*	IRb	143240	144033	232	536	26		
*clpP*	LSC	71714	73781	228	668	289	812	71
*petB*	LSC	76703	78109	6	759	642		
*petD*	LSC	78297	79522	8	743	475		
*rpl16*	LSC	83036	84454	399	1011	9		
*rpl2*	IRa	86241	87742	434	683	385		
*ndhB*	IRa	96877	99089	756	680	777		
*ndhA*	SSC	122795	125028	539	1142	553		
*ndhB*	IRb	144889	147101	777	680	756		
*rpl2*	IRb	156236	157737	385	683	434		

### Comparative plastome structure and polymorphism

**Fig 2:**
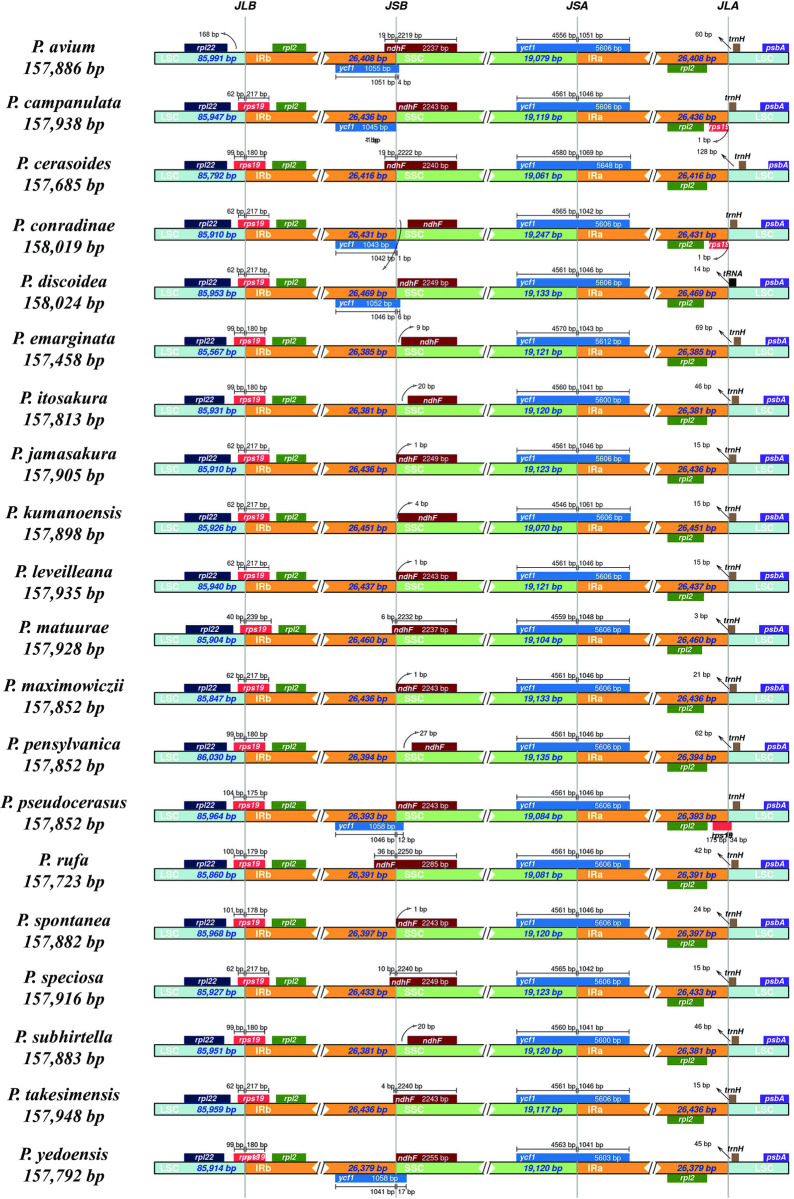
Comparisons of LSC, SSC and IR region boundaries among the plastomes of the 20 subg. *Cerasus* species studied.

Though the plastome is usually well conserved, contraction and expansion of IR regions is the main cause of plastome differences among different plant species [13]. The IR and SC boundaries of 20 subg. *Cerasus* species are shown in Fig 2. The gene arrangement and content of these species are similar in the IR/SC boundaries, which were located in the *rps19* and *ycf1* genes. The *rps19* gene, positioned within the boundary of the LSC/IRb region of these species except for *P*. *avium*, showed the same fragment size of 279 bp in all species (Fig 2). The fragment size ranges from 175 to 239 bp in the IRb region while the tail section of the gene located in the LSC region ranged from 40 to 104 bp (Fig 2). The *ycf1* gene is a critical gene that crossed the SSC/IRa border with 4,546–4,580 bp in the SSC regions and 1,041–1,069 bp in the IRa regions. The *ndhF* gene ranged from 2,237 to 2,285 bp and was located in the boundary of the IRb/SSC region in these tested species. However, the whole *ndhF* gene was situate in the SSC region in *P*. *campanulata*, *P*. *conradinae*, *P*. *discoidea*, *P*. *emarginata*, *P*. *itosakura*, *P*. *jamasakura*, *P*. *kumanoensis*, *P*. *leveilleana*, *P*. *maximowiczii*, *P*. *pensylvanica*, *P*. *pseudocerasus*, *P*. *spontanea*, *P*. *subhirtella* and *P*. *yedoensis* (Fig 2). In addition, the *trnH* gene was located in the LSC region of all tested species, with the distance of the *trnH* gene from the IRa/SSC boundary ranging from 1 to 128bp. Furthermore, the *rps19* pseudogenes spanned two regions in *P*. *campanulata*, *P*. *conradinae* and *P*. *pseudocerasus* at the IRa/LSC boundary, with fragment sizes of 217, 217 and 210 bp, respectively (Fig 2). The *ycf1* pseudogene with similar fragment size across the IRb/SSC boundary was also found in *P*. *avium*, *P*. *campanulata*, *P*. *conradinae*, *P*. *discoidea*, *P*. *pseudocerasus*, and *P*. *yedoensis*. The pseudogene fragment sizes of ycf1 in the above different species are 1,055, 1,045, 1,043, 1,052, 1,058, and 1,058 bp, respectively (Fig 2). By comparing the IR and SC regions, it is clear that the full-length differences of the 20 subg. *Cerasus* plastome sequences are caused by the differences in the size of the genes at the IR/SC boundaries.

**Fig 3:**
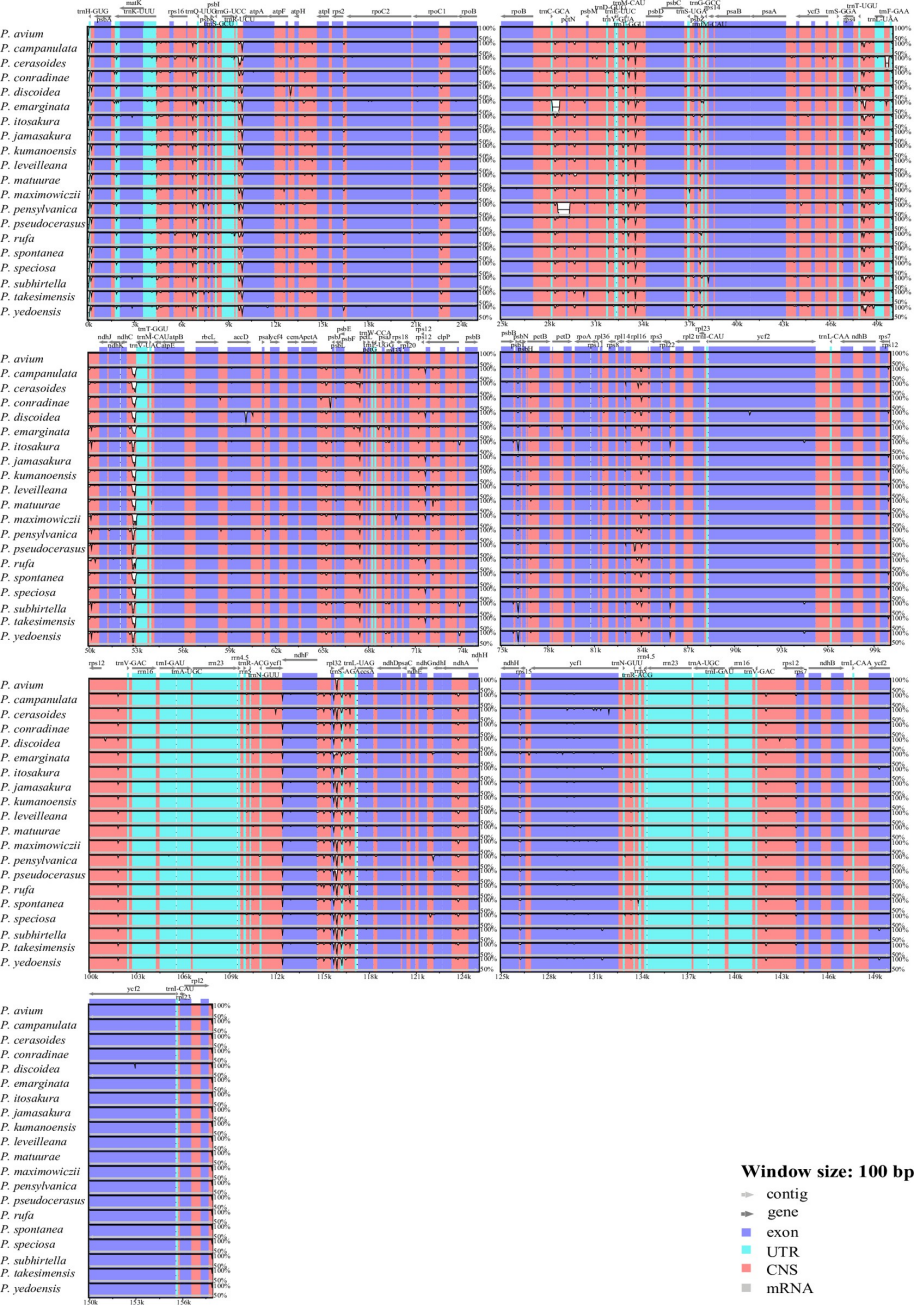
Sequence alignment of 20 subg. *Cerasus* plastomes with *P*. *avium* as the reference. The y-axis represents the percent similarity between 50% and 100%. Different colors represent different genetic regions.

The Shuffle-LAGAN mode of mVISTA online software was employed to compare the sequence discrepancy of 20 plastomes of subg. *Cerasus* with the sequence of *P*. *avium* as reference (Fig 3). The plastome alignments were more conserved in the coding regions than the non-coding regions. Moreover, there was more variation in the intergenic spacer (IGS) regions: *trnK-rps16*, *rps16-trnQ*, *trnR-atpA*, *atpF-atpH*, *trnC-petN*, *trnT-psbD*, *trnT-trnL*, *ndhC-trnV*, *petA-psbJ*, *psbF-petL*, *rpl16-rps3*, *rpl32-trnS*, *trnS-trnL* (Fig 3). In addition, the coding regions with slight variation contained *accD*, *psbN*, *ndhF*, *rpl32*, *trnS*, *rps15* and *ycf1* (Fig 3). *P*. *emarginata* and *P*. *pensylvanica* exhibited polymorphisms on the intergenic region between *trnC* and *petN*, which were mainly manifested in the deletion of partial sequence fragments.

We identified a total of 99 shared genes and IGS regions from the plastomes of the 20 subg. *Cerasus* species studied (see S1 Table and S1 Fig). The nucleotide diversity (Pi) of the coding sequence (CDS) ranged from 0 to 0.05526 and the IGS ranged from 0.00070 to 0.00822. Among these, tRNA *trnG-GCC* had the most nucleotide diversity (0.05526), while the protein coding gene *ndhI*, which is located in the LSC and SSC region, has the most nucleotide variation (0.00414). The IGS regions *rpl32-trnL* and *trnR-atpA* showed highly variable polymorphism (Pi > 0.05), with the values of nucleotide diversity 0.00822 and 0.00567, respectively (S1 Table). Therefore, these four sequences could be used to develop useful makers for phylogenetic analysis and distinguishing taxa in subg. *Cerasus*.

### Codon usage pattern

According to the codon usage analysis, overall 64 codons that encode 20 amino acids (AAs) are present across the 20 subg. *Cerasus* species studied. The protein-coding sequences of the plastomes of the 20 species consist of 26158, 26061, 26516, 26172, 26490, 26525, 26163, 26152, 26160, 26158, 26156, 26158, 26162, 26680, 26171, 26158, 26164, 26152, 26158, 26565 codons, respectively (S2 Table). Among the encoded amino acids, leucine is most frequent and cysteine least frequent. Of the 20 subg. *Cerasus* species studied, *P*. *campanulata* and *P*. *pseudocerasus* have 31 codons less frequently used than the expected usage at equilibrium (RSCU < 1) while the other 18 subg. *Cerasus* taxa showed codon usage bias (RSCU < 1) in 32 codons. All 20 subg. *Cerasus* species had 30 codons more frequently used than the expected usage at equilibrium (RSCU >1). Codons with A and/or U in the third position take up ~43% and ~47% of all codons respectively (S2 Table). The frequency of use for the start codons AUG and UGG, encoding methionine and tryptophan, showed no bias (RSCU = 1) in all tested species. The codon UCC encoding serine in *P*. *campanulata* and *P*. *pseudocerasus* also showed no bias (RSCU = 1) (S2 Table).

### Tandem repeats and simple sequence repeats (SSRs)

SSRs, also called microsatellites, were extensively distributed in the plastome of all species studied. Using MISA software, we found the total number of SSRs in the 20 subg. *Cerasus* species range from 70 to 94 (S3 Table and S2 Fig). Of the mono-, di-, tri-, tetra-, penta-, and hexa-nucleotide categories of SSRs present in the plastomes of the subg. *Cerasus* taxa, mononucleotide repeats are the most abundant, ranging from 60.00% (*P*. *yedoensis*) to 71.27% (*P*. *leveilleana*). Pentanucleotide repeats were detected in *P*. *avium*, *P*. *kumanoensis*, *P*. *rufa*, *P*. *speciose*, *P*. *subhirtella*, *P*. *takesimensis* and *P*. *yedoensis*, which accounts for the low proportion of SSRs in these seven species. Except for *P*. *campanulata*, *P*. *subhirtella* and *P*. *yedoensis*, the rest of these species all contain hexanucleotide repeats. Among all tested subg. *Cerasus* taxa, only one trinucleotide repeat is detected in *P*. *pseudocerasus*.

**Fig 4:**
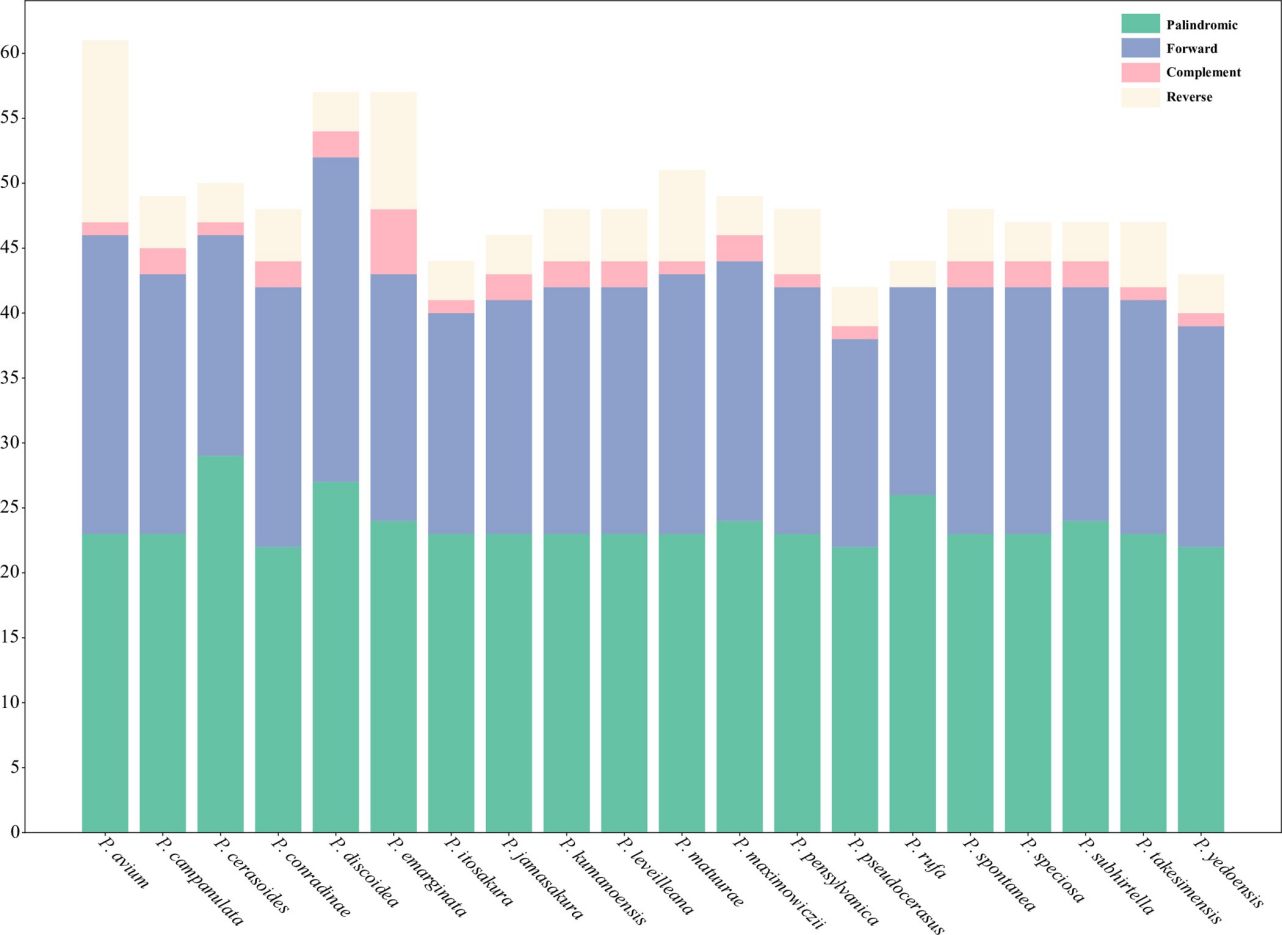
Investigation of repeated sequences in subg. *Cerasus* plastomes. The 20 subg. *Cerasus* plastomes studied have four repeat types, which are forward (F), reverse (R), palindrome (P) and complement (C).

Different types of compound SSRs were detected across the 20 subg. *Cerasus* species studied. Of all the subg. *Cerasus* species studied, *P*. *avium* contained the largest number of repeat types (Fig 4 and S4 Table). Among the four repeat types known, the most common repeat type is palindromic repeats, which ranged from 37.70% in *P*. *avium* to 59.09% in *P*. *rufa*, which explains why *P*. *rufa* contains no complement repeats.

### Phylogenetic analysis

**Fig 5:**
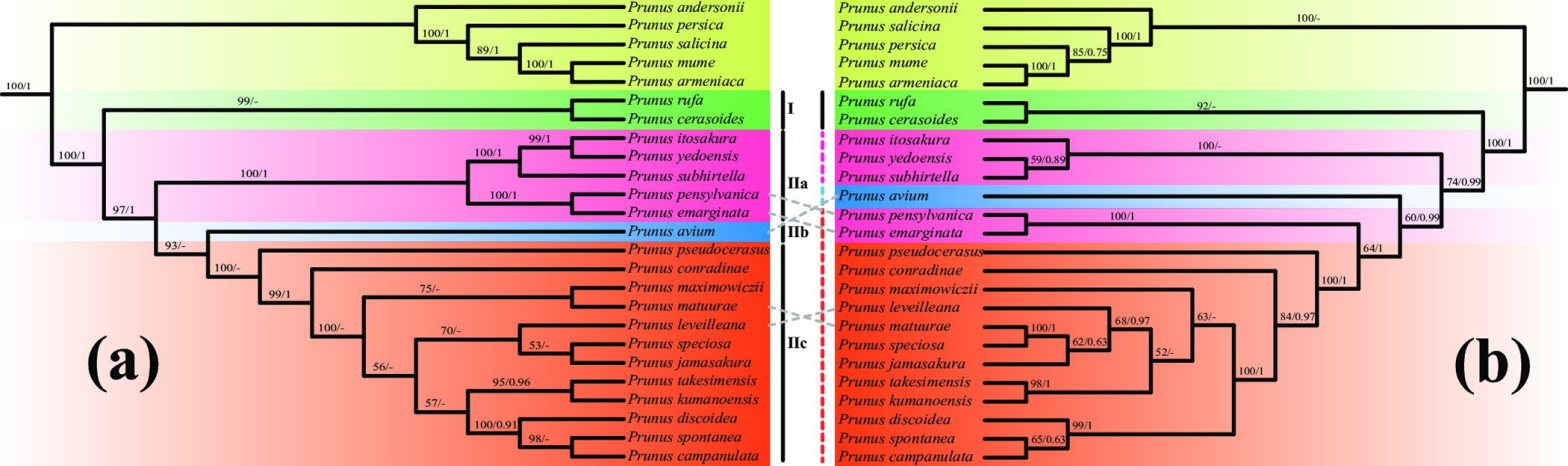
Combined ML and BI phylogenetic trees of 20 subg. *Cerasus* species based on either (a) complete plastome data or (b) coding region (CDS) data. The support value is displayed above the branch in the order of Maximum Likelihood bootstrap support and Bayesian Inference posterior probability. “‐” indicates the branch collapse in the Bayesian tree. Clades, subclades or lineages are indicated by gradual colors of boxes covered on taxa names, taxa with changing molecular placement are connected by dotted lines.

In order to resolve the phylogenetic placement of taxa of subg. *Cerasus* in *Prunus s*.*l*. and interspecific relationships within subg. *Cerasus*, we used Maximum Likelihood (ML) analysis and Bayesian Inference (BI) analyses to perform phylogenetic analyses using the complete plastome data and CDS regions of 53 published complete plastid sequences (S5 Table and S3 Fig). Similar phylogenetic topologies were obtained in both ML and BI analyses. In these topologies, *Prunus* taxa formed a monophyletic group which diverged into two major clades with strong bootstrap support, with one clade formed of all 20 subg. *Cerasus* taxa and the other clade of 5 other *Prunus* taxa belonging to *Prunus s*.*l*. The 20 subg. *Cerasus* taxa formed a monophyletic group that were divided, albeit on very short branch lengths but with high support, into two principal clades (Figs 5 and S3). Clade Ⅰ was basal in the monophyly of subg. *Cerasus* and contained *P*. *cerasoides* and *P*. *rufa* from central and southern Asia. Clade Ⅱ was further divided, again on very short branch lengths and with mixed support, into three sub-clades. Clade Ⅱb contained only one taxa *P*. *avium*, with high support in the ML tree on plastome data but weak support in trees run with CDS data, while the branch collapsed in BI analyses.

When comparing topologies based on just plastome data or CDS regions, Clade Ⅱa and Clade Ⅱc exhibited differences (Fig 5). Using just plastome data (Fig 5A), Clade Ⅱc included 12 taxa, while when using data from just CDS regions (Fig 5B) it contained 14 taxa, with the additional two species, *P*. *emarginata* and *P*. *pensylvanica*. In both topologies, *P*. *emarginata* and *P*. *pensylvanica* are shown to be closely related and forming a clade, but the position of this clade, whether in Clade IIa or IIc, is unresolved. In addition, *P*. *matuurae* exhibits a closer relationship with *P*. *speciosa* in the phylogenetic trees constructed with CDS regions (Fig 5B), while the topology constructed on plastome data places it in a calde with *P*. *maximowiczii* (Fig 5A). In both topologies, a number of moderate or strongly supported relationships were retrieved, albeit with short branch lengths: a) *P*. *itosakura*, *P*. *yedoensis* and *P*. *subhirtella* formed a clade in Clade Ⅱa; b) *P*. *pseudocerasus* is positioned at the basal node in Clade Ⅱc; c) *P*. *kumanoensis* and *P*. *takesimensis* formed a sub-clade in Clade Ⅱc; d) *P*. *campanulata*, *P*. *spontanea* and *P*. *discoidea* formed a sub-clade in Clade Ⅱc.

## Discussion

### Variations in plastome structure

Analyzing the genetic background and diversity of Subg. *Cerasus* is a challenge. The phylogenetic, diversity, and genetic relationships based on simple chloroplast markers and sequence data were previously reported in Subg. *Cerasus* [8,9].

The plastomes of angiosperms exhibit a highly conserved structure and organization of gene content [26,27]. The subg. *Cerasus* plastomes also exhibit the typical quadripartite structure and similar complete plastome sequence size of angiosperms, ranging from 157,458 bp in *P*. *emarginata* to 158,024 bp in *P*. *discoidea* (Table 1 and Fig 1). Previous studies on angiosperms have revealed that the number of genes in plastomes ranges from 120 to 130 [26]. However, in this study, subg. *Cerasus* plastomes contained 134 genes, including 78 protein coding genes, 31 tRNA- and 4 rRNA-coding genes (Table 2). The plastomes among these species were similar in intron and GC contents (Tables 1 and 3). The number of genes containing introns was 12, suggesting that the intron contents in subg. *Cerasus* are also similar to those of most flowering plant clades [16]. However, the GC content in the IR regions was significantly higher than that in the LSC and SSC regions. The main reason for this is that all eight rRNA genes with high GC contents are distributed in the IR region. In general, the IR region is the most conserved region of the plastome [15], and expansion and contraction of the IR regions are the main causes of different plastome lengths [13,28]. In this study, we found only small changes in the organization of genes in the IR region and boundary between the IR and LSC and SSC regions in the 20 plastomes studied. The distribution of genes across the SC-IR boundaries, such as *rps19*, *ndhF*, *ycf1*, etc. are similar to that of other *Prunus* species [15] (Fig 2).

### Variation of SSRs

SSRs are highly polymorphic and codominantly inherited, and are seen as valuable markers for studies involving gene diversity, population genetics and gene mapping. Previous research found that SSRs can be widely used as important resources of molecular markers and these have been broadly applied in phylogenetic and biogeographic studies [29,30]. We counted the quantities of SSRs for the 20 species in subg. *Cerasus*, with the largest number of SSR types being mononucleotide repeats, which is consistent with results from other studies of angiosperms [12,15,16]. In the plastomes of subg. *Cerasus* taxa, the number of SSRs was found to be significantly higher than that in other angiosperms, and the content of A/T repeats is far greater than that of G/C repeats, similar to the results of Melotto-Passarin and other studies [31,32] (S3 Table).

### Polymorphism in IGS and CDS

Additionally, we also analyzed the sequence polymorphism of these 20 subg. *Cerasus* taxa in both IGS and CDS regions (S1 Table and S1 Fig). Consistent with the diversity patterns found in most angiosperms [16,33,34], sequence divergence in IGS regions was higher than that in CDS regions. We then calculated the Pi value of the coding genes and six IGS regions in the complete sequence and screened out the Pi value more than 0.05. Among these sequences, *trnG-GCC*, *ndhI*, *rpl32*-*trnL* and *trnR*-*atpA* have been previously known as hypervariable regions in *Prunus* species [15], and we speculate that these fragments with high polymorphism can be used as hot spots for developing molecular markers, allowing a more efficient study of phylogenetic relationships within subg. *Cerasus*.

### Phylogenetic relationships

Both molecular and morphological evidence suggests that subg. *Cerasus* had a complex evolutionary history. In order to obtain a clearer understanding of phylogenetic relationships within Rosaceae and *Prunus s*.*l*., previous studies selected either a few gene fragments or the complete plastome to reconstruct the phylogenetic framework [7–9,35–40].

Our study indicates that taxa of subg. *Cerasus* with corymbs are monophyletic and with a clear sister relationship to other single-flowered and racemose groups within *Prunus s*.*l*. (Figs 5 and S3). Our selection of outgroups for the phylogenetic analysis was based on the large-scale phylogenomic study of Rosaceae [7], with the topological structure retrieved in this study similar to our own. These similarities include high support for a sister relationship of Amygdaleae and Sorbarieae (S3 Fig) and subg. *Cerasus* being positioned in Amygdaleae as a monophyletic group. This result is also consistent with other previous studies on the phylogeny of *Prunus s*.*l*. [8].

Plastid phylogenomics provides one possible solution for studies focused on problematic phylogenetic relationships [41]. The relationships between taxa of subg. *Cerasus* retrieved in our study differed from previous studies that did not incorporate plastid phylogenomics. For example, Shi et al. found *P*. *avium* and *P*. *pseudocerasus* to be closely related, while our study suggests *P*. *avium* to be basally positioned (Clade IIb) in the monophyletic subg. *Cerasus* clade, with *P*. *pseudocerasus* placed basal in Clade IIc [8]. In their study, subg. *Cerasus* was divided into two clades. However, the phylogenetic topology in our study based on two methods (ML and BI) found that subg. *Cerasus* was also divided into two major clades with high support but a short branch length. Clade Ⅰ contains *P*. *cerasoides* and *P*. *rufa*, with a central and southern Asian distribution.

Placement of certain taxa within Clade II was also unresolved due to inconsistencies when comparing topologies based on complete plastome or CDS datasets. This includes the *P*. *emarginata* + *P*. *pensylvanica* clade being placed in Clade IIa or IIc, depending on complete plastome or CDS data, respectively. CDS data also provided greater resolution on certain relationships, such as placing *P*. *matuurae* with *P*. *speciosa* in a clade that is rooted in a large polytomy in clade IIc. While the topology based on complete plastome shows no such relationship, with both species held in the large polytomy. The causes of the insignificant divergence of subg. *Cerasus* taxa may be due to quantum speciation or low resolution of the markers. Widespread interspecific hybridisations also have complicated the taxonomy of this subgenus [6].

Nevertheless, certain relationships in Clade II were well-supported and consistent. Despite an unresolved placement within Clade II, the relationship between *P*. *emarginata* and *P*. *pensylvanica* was notable, with these species sharing similar geographical distribution and habitat in North American. High support for the basal position of *P*. *pseudocerasus* in Clade IIc was also found in all analyses. *Prunus campanulata*, *P*. *spontanea* and *P*. *discoidea* formed a sub-clade in Clade Ⅱc. *P*. *kumanoensis* and *P*. *takesimensis*, these species with an eastern Asian distribution, also consistently formed a well-supported sub-clade in Clade Ⅱc in all analyses. While their relationship with *P*. *emarginata* and *P*. *pensylvanica* is still unresolved, *P*. *itosakura*, *P*. *yedoensis* and *P*. *subhirtella* showed a well-supported relationship and were consistently grouped in Clade Ⅱa.

## Conclusions

In this study, we revealed the plastome size, GC content, and gene number and order among 20 subg. *Cerasus* species. We identified highly polymorphic regions of nucleotide variation that are potential molecular markers for identifying and overcoming phylogenetic problems at the species level. We found that SSRs in our plastomes might also provide intraspecific level polymorphic markers that can be used for population genetic studies.

The phylogenetic trees constructed based on complete plastome and CDS regions all exhibited similar topologies, albeit with certain irregularities and discordances and a lack of resolution within certain clades. We find support for *P*. *cerasoides* and *P*. *rufa* as basal in the monophyly of subg. *Cerrasus*, as well as a close relationship between certain groups of taxa. Undoubtedly, taxa of subg. *Cerasus* are a monophyletic group sister to taxa of *Prunus s*.*l*., but some infrageneric relationships within subg. *Cerasus* remain unresolved, as can be seen by certain low bootstrap values and changing positions of taxa depending on datasets used. Thus, it is particularly important to explore characteristics of repeat elements and in-depth information mining of plastomes in future research as well as inclusion of greater taxon sampling to help clarify these relationships.

## Supporting information

S1 Fig Comparative analysis of the nucleotide diversity by Pi values of the 20 subg. *Cerasus* species studied. (PDF)

S2 Fig Number of motif types in SSRs in 20 subg. *Cerasus* complete plastomes. (PDF)

S3 Fig Combined ML and BI phylogenetic trees of 20 subg. *Cerasus* species based on either (a) complete plastome data or (b) coding region (CDS) data. The support value is displayed above the branch in the order of Maximum Likelihood bootstrap support and Bayesian Inference posterior probability. “‐” indicates the branch collapse in the Bayesian tree.
(PDF)

S1 Table The statistics of nucleotide diversity (Pi) between the species of 20 subg. *Cerasus* chloroplast genome. (DOCX)

S2 Table Codon features of 20 Subg. *Cerasus* complete plastomes. (DOCX)

S3 Table Number of analyses of simple sequences repeats (SSRs) in 20 subg. *Cerasus* complete plastomes. (DOCX)

S4 Table The statistics of four repeat types in 20 subg. *Cerasus* complete plastomes. (DOCX)

S5 Table Summary for all the information of 33 species. (DOCX)
