# Supplementary figures and images for: Comparative analysis of the complete plastid genomes in Prunus subgenus Cerasus (Rosaceae): Molecular structures and phylogenetic relationships
Source: PLoS One. 2022 Apr 6;17(4):e0266535. doi: 10.1371/journal.pone.0266535 (PMC8985974; doi:10.1371/journal.pone.0266535)

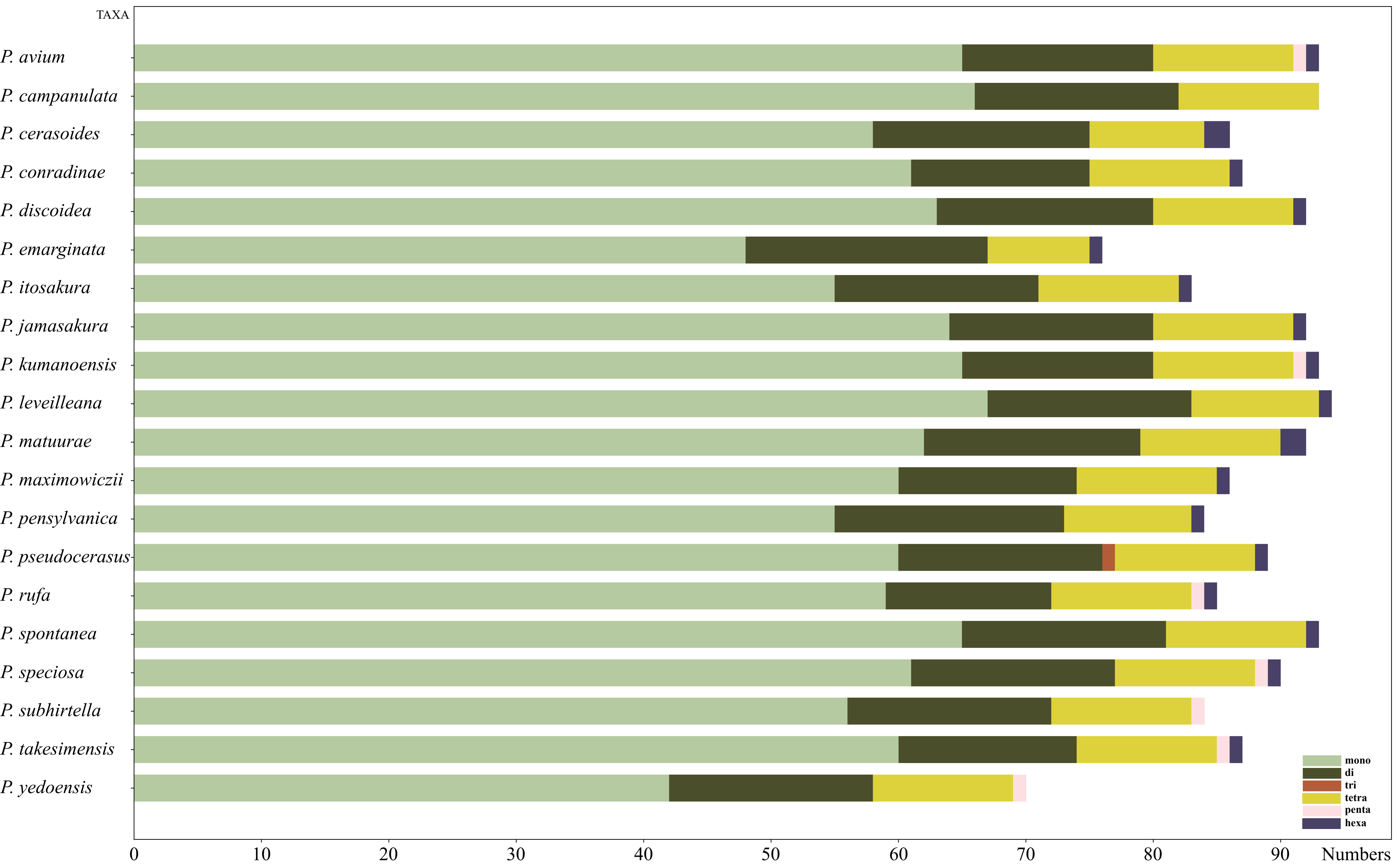

Supplement: S2 Fig — (PDF) [file pone.0266535.s002.pdf]

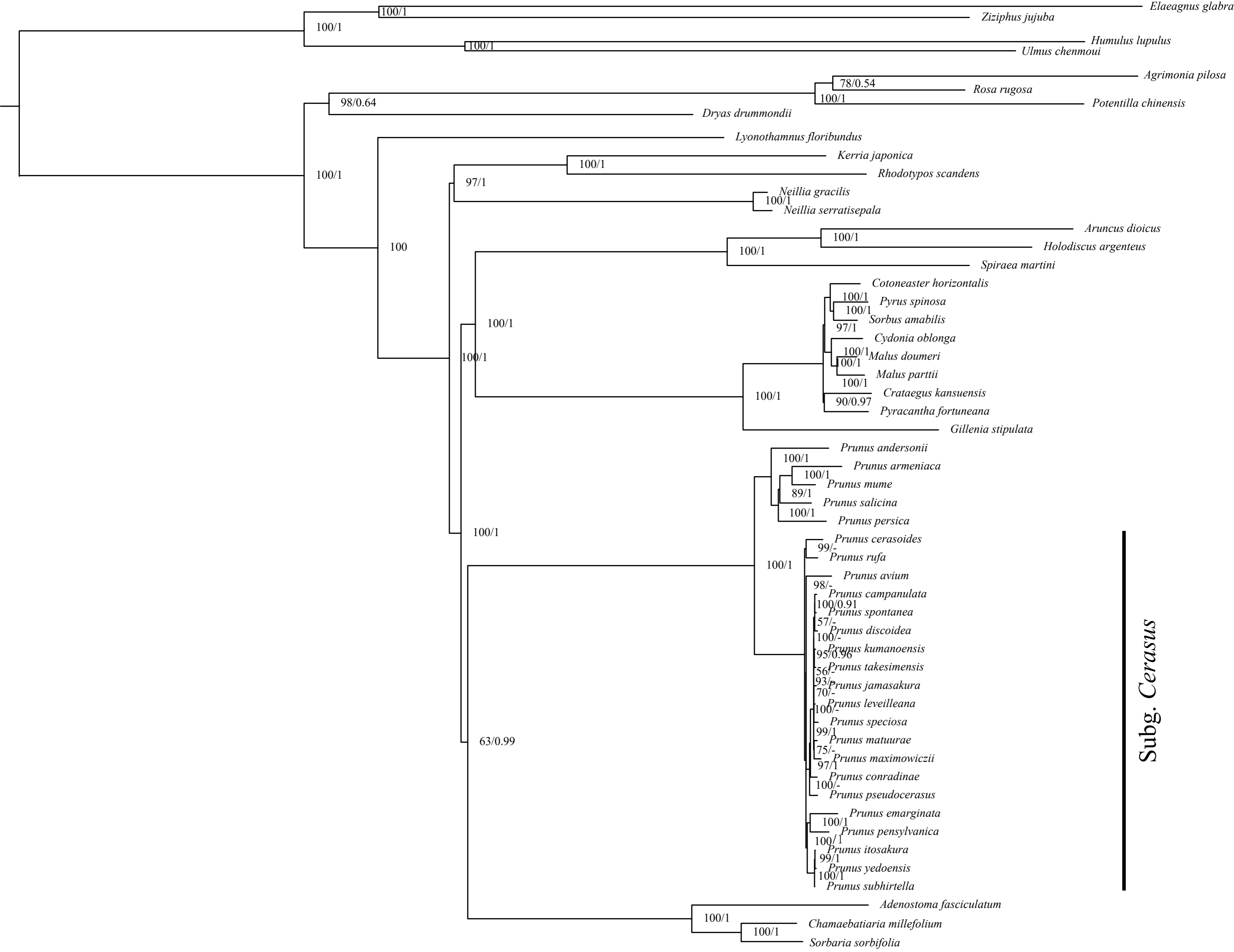

(a)

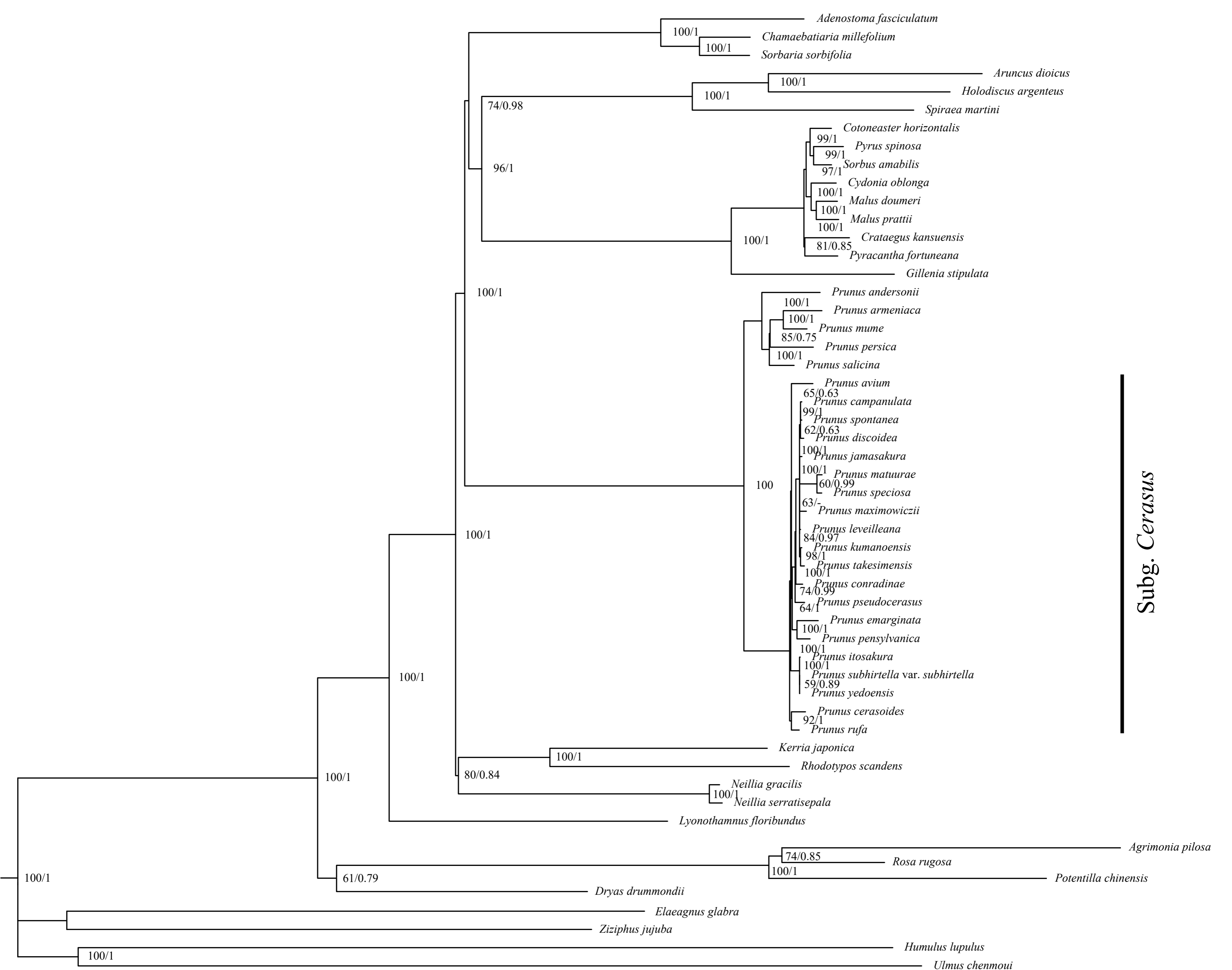

(b)

Supplement: S3 Fig — Combined ML and BI phylogenetic trees of 20 subg. Cerasus species based on either (a) complete plastome data or (b) coding region (CDS) data. The support value is displayed above the branch in the order of Maximum Likelihood bootstrap support and Bayesian Inference posterior probability. “‐” indicates the branch collapse in the Bayesian tree. (PDF) [file pone.0266535.s003.pdf]
