# Supplementary material for: Comparative analysis of the complete plastid genomes in Prunus subgenus Cerasus (Rosaceae): Molecular structures and phylogenetic relationships
Source: PLoS One. 2022 Apr 6;17(4):e0266535. doi: 10.1371/journal.pone.0266535 (PMC8985974; doi:10.1371/journal.pone.0266535)
Supplement: S1 Table — (DOCX) [file pone.0266535.s004.docx]

**S1 Table.** The statistics of nucleotide diversity (Pi) between the species of 20 subg. *Cerasus* chloroplast genome.

| **gene** | **Pi value** | **gene** | **Pi value** | **gene** | **Pi value** |
| --- | --- | --- | --- | --- | --- |
| *psbA* | 0.000377 | *rbcL* | 0.000468 | *ndhB* | 0.000176 |
| *matK* | 0.001963 | *accD* | 0.001102 | *rps7* | 0.000214 |
| *rps16* | 0.001464 | *psaI* | 0.000877 | *rrn16* | 0 |
| *psbK* | 0.000556 | *ycf4* | 0.000541 | *trnI-GAU* | 0.000382 |
| *psbI* | 0 | *cemA* | 0.001144 | *trnA-UGC* | 0.000114 |
| *atpA* | 0.000608 | *petA* | 0.000404 | *rrn23* | 0 |
| *atpF* | 0.001535 | *psbJ* | 0 | *rrn4.5* | 0 |
| *atpH* | 0.000407 | *psbL* | 0 | *rrn5* | 0 |
| *atpI* | 0.000523 | *psbF* | 0.000833 | *trnN-GUU* | 0 |
| *rps2* | 0.000844 | *psbE* | 0.001859 | *ndhF* | 0.001649 |
| *rpoC2* | 0.00087 | *petL* | 0 | *rpl32* | 0.001192 |
| *rpoC1* | 0.001194 | *petG* | 0 | *ccsA* | 0.001276 |
| *rpoB* | 0.000516 | *psaJ* | 0.000741 | *ndhD* | 0.000795 |
| *trnC-GCA* | 0 | *rpl33* | 0.000498 | *psaC* | 0.000407 |
| *petN* | 0 | *rps18* | 0 | *ndhE* | 0.00098 |
| *psbM* | 0.001805 | *rpl20* | 0.003063 | *ndhG* | 0.000377 |
| *trnY-GUA* | 0 | *rps12* | 0.000269 | *ndhI* | 0.00414 |
| *trnE-UUC* | 0 | *clpP* | 0.001215 | *ndhA* | 0.00124 |
| *trnM-CAU* | 0.018644 | *psbB* | 0.000321 | *ndhH* | 0.001015 |
| *psbD* | 0 | *psbT* | 0 | *rps15* | 0.000733 |
| *psbC* | 0.000133 | *psbN* | 0 | *ycf1* | 0.000746 |
| *psbZ* | 0 | *psbH* | 0.000444 | *trnR-ACG* | 0 |
| *trnG-GCC* | 0.055263 | *petB* | 0.001902 | *ycf2* | 0.000161 |
| *rps14* | 0.00099 | *petD* | 0.001321 | *ndhC_trnV* | 0.002754237 |
| *psaB* | 0.000561 | *rpoA* | 0.000907 | *rpl32_trnL* | 0.008223684 |
| *ycf3* | 0.000455 | *rps11* | 0 | *trnC_petN* | 0.000700935 |
| *rps4* | 0.000773 | *rpl36* | 0 | *trnR_atpA* | 0.005671278 |
| *trnL-UAA* | 0.002764 | *rps8* | 0 | *trnS_trnG* | 0.003489386 |
| *trnF-GAA* | 0 | *rpl14* | 0.000271 | *trnT_trnL* | 0.004704133 |
| *ndhJ* | 0.000629 | *rpl16* | 0.000859 |  |  |
| *ndhK* | 0.001167 | *rps3* | 0.000609 |  |  |
| *ndhC* | 0.000551 | *rpl22* | 0.000704 |  |  |
| *trnV-UAC* | 0.000602 | *rpl2* | 0.000333 |  |  |
| *atpE* | 0.001794 | *rpl23* | 0 |  |  |
| *atpB* | 0.00152 | *trnL-CAA* | 0 |  |  |
