# Supplementary material for: Comparative analysis of the complete plastid genomes in Prunus subgenus Cerasus (Rosaceae): Molecular structures and phylogenetic relationships
Source: PLoS One. 2022 Apr 6;17(4):e0266535. doi: 10.1371/journal.pone.0266535 (PMC8985974; doi:10.1371/journal.pone.0266535)
Supplement: S2 Table — (DOCX) [file pone.0266535.s005.docx]

**S2 Table.** Codon features of 20 Subg. *Cerasus* chloroplast genomes.

| **Species** | **coding genes codons** | **A3s** | **U3s** | **RSCU** | | |
| --- | --- | --- | --- | --- | --- | --- |
|  |  |  |  | **>1** | **<1** | **=1** |
| *Prunus avium* | 26061 | 0.4338 | 0.467 | 30 | 32 | 2 |
| *Prunus campanulata* | 26516 | 0.4341 | 0.4662 | 30 | 31 | 3 |
| *Prunus cerasoides* | 26172 | 0.4341 | 0.4671 | 30 | 32 | 2 |
| *Prunus conradinae* | 26490 | 0.4342 | 0.466 | 30 | 32 | 2 |
| *Prunus discoidea* | 26525 | 0.4338 | 0.4659 | 30 | 32 | 2 |
| *Prunus emarginata* | 26163 | 0.434 | 0.4672 | 30 | 32 | 2 |
| *Prunus itosakura* | 26152 | 0.4338 | 0.4667 | 30 | 32 | 2 |
| *Prunus jamasakura* | 26160 | 0.4339 | 0.4666 | 30 | 32 | 2 |
| *Prunus kumanoensis* | 26158 | 0.434 | 0.4666 | 30 | 32 | 2 |
| *Prunus leveilleana* | 26158 | 0.434 | 0.4666 | 30 | 32 | 2 |
| *Prunus matuurae* | 26156 | 0.4333 | 0.4668 | 30 | 32 | 2 |
| *Prunus maximowiczii* | 26158 | 0.4339 | 0.4665 | 30 | 32 | 2 |
| *Prunus pensylvanica* | 26162 | 0.4343 | 0.4667 | 30 | 32 | 2 |
| *Prunus pseudocerasus* | 26680 | 0.4341 | 0.4658 | 30 | 31 | 3 |
| *Prunus rufa* | 26171 | 0.434 | 0.4669 | 30 | 32 | 2 |
| *Prunus spontanea* | 26158 | 0.4338 | 0.4665 | 30 | 32 | 2 |
| *Prunus speciosa* | 26164 | 0.4336 | 0.4669 | 30 | 32 | 2 |
| *Prunus subhirtella* | 26152 | 0.4337 | 0.4667 | 30 | 32 | 2 |
| *Prunus takesimensis* | 26158 | 0.4339 | 0.4666 | 30 | 32 | 2 |
| *Prunus yedoensis* | 26565 | 0.4342 | 0.4662 | 30 | 32 | 2 |
| Average |  | 0.4339 | 0.4665 |  |  |  |
