# Supplementary material for: Comparative analysis of the complete plastid genomes in Prunus subgenus Cerasus (Rosaceae): Molecular structures and phylogenetic relationships
Source: PLoS One. 2022 Apr 6;17(4):e0266535. doi: 10.1371/journal.pone.0266535 (PMC8985974; doi:10.1371/journal.pone.0266535)
Supplement: S3 Table — (DOCX) [file pone.0266535.s006.docx]

Table S3 Number of analyses of simple sequences repeats (SSRs) in 20 subg. *Cerasus* complete plastomes

| **Species** | **SSR types** | | | | | | | | | | | | | |
| --- | --- | --- | --- | --- | --- | --- | --- | --- | --- | --- | --- | --- | --- | --- |
|  | **A/T** | **C/G** | **AG/CT** | **AT/AT** | **AAT/ATT** | **AAAG/CTTT** | **AAAT/ATTT** | **AATC/ATTG** | **AATT/AATT** | **AATAT/ATATT** | **AAATT/AATTT** | **AAATAT/ATATTT** | **AAATTT/AAATTT** |  |
| *Prunus avium* 'summit' | 53 | 1 | — | 15 | — | 1 | 7 | 1 | 2 | 1 | — | 1 | — |  |
| *Prunus campanulata* | 65 | 1 | 1 | 15 | — | 1 | 7 | 1 | 2 | — | — | — | — |  |
| *Prunus cerasoides* | 57 | 1 | 1 | 16 | — | 1 | 5 | 1 | 2 | — | — | 1 | 1 |  |
| *Prunus conradinae* | 59 | 2 | 1 | 13 | — | 1 | 7 | 1 | 2 | — | — | 1 | — |  |
| *Prunus discoidea* | 62 | 1 | 1 | 16 | — | 1 | 7 | 1 | 2 | — | — | 1 | — |  |
| *Prunus emarginata* | 48 | 1 | — | 18 | — | 1 | 5 | 1 | 1 | — | — | 1 | — |  |
| *Prunus itosakura* | 54 | 1 | 1 | 15 | — | 1 | 7 | 1 | 2 | — | — | 1 | — |  |
| *Prunus jamasakura* | 63 | 1 | 1 | 15 | — | 1 | 7 | 1 | 2 | — | — | 1 | — |  |
| *Prunus kumanoensis* | 64 | 1 | — | 15 | — | 1 | 7 | 1 | 2 | 1 | — | 1 | — |  |
| *Prunus leveilleana* | 66 | 1 | 1 | 15 | — | 1 | 6 | 1 | 2 | — | — | 1 | — |  |
| *Prunus matuurae* | 60 | 2 | 1 | 16 | — | 1 | 7 | 1 | 2 | — | — | 2 | — |  |
| *Prunus maximowiczii* | 59 | 1 | 1 | 13 | — | 1 | 7 | 1 | 2 | — | — | 1 | — |  |
| *Prunus pensylvanica* | 54 | 1 | 1 | 17 | — | 1 | 6 | 1 | 2 | — | — | 1 | — |  |
| *Prunus pseudocerasus* | 59 | 1 | 1 | 15 | 1 | 1 | 7 | 1 | 2 | — | — | 1 | — |  |
| *Prunus rufa* | 58 | 1 | 1 | 12 | — | 1 | 7 | 1 | 2 | — | 1 | 1 | — |  |
| *Prunus serrulata var. spontanea* | 64 | 1 | 1 | 15 | — | 1 | 7 | 1 | 2 | — | — | 1 | — |  |
| *Prunus speciosa* | 60 | 1 | 1 | 15 | — | 1 | 7 | 1 | 2 | 1 | — | 1 | — |  |
| *Prunus subhirtella* var. *subhirtella* | 55 | 1 | 1 | 15 | — | 1 | 7 | 1 | 2 | — | 1 | — | — |  |
| *Prunus yedoensis* | 59 | 1 | — | 14 | — | 1 | 7 | 1 | 2 | 1 | — | 1 | — |  |
| *Prunus yedoensis* | 41 | 1 | 1 | 15 | — | 1 | 7 | 1 | 2 | — | 1 | — | — |  |
