# Supplementary material for: Comparative analysis of the complete plastid genomes in Prunus subgenus Cerasus (Rosaceae): Molecular structures and phylogenetic relationships
Source: PLoS One. 2022 Apr 6;17(4):e0266535. doi: 10.1371/journal.pone.0266535 (PMC8985974; doi:10.1371/journal.pone.0266535)
Supplement: S4 Table — (DOCX) [file pone.0266535.s007.docx]

Table S4 The statistics of four repeat types in 20 subg. *Cerasus* chloroplast genome

| **Species** | **P** | **F** | **C** | **R** | **Total** | **NCBI accession number** | **URL** |
| --- | --- | --- | --- | --- | --- | --- | --- |
| *Prunus avium* | 23 | 23 | 1 | 14 | 61 | NC044701 | https://www.ncbi.nlm.nih.gov/nuccore/NC_044701 |
| *Prunus campanulata* | 23 | 20 | 2 | 4 | 49 | NC044123 | https://www.ncbi.nlm.nih.gov/nuccore/NC_044123 |
| *Prunus cerasoides* | 29 | 17 | 1 | 3 | 50 | NC035891 | https://www.ncbi.nlm.nih.gov/nuccore/NC_035891 |
| *Prunus conradinae* | 22 | 20 | 2 | 4 | 48 | MT374065 | https://www.ncbi.nlm.nih.gov/nuccore/MT374065 |
| *Prunus discoidea* | 27 | 25 | 2 | 3 | 57 | MN158647 | https://www.ncbi.nlm.nih.gov/nuccore/MN158647 |
| *Prunus emarginata* | 24 | 19 | 5 | 9 | 57 | MN389436 | https://www.ncbi.nlm.nih.gov/nuccore/MN389436 |
| *Prunus itosakura* | 23 | 17 | 1 | 3 | 44 | MN695296 | https://www.ncbi.nlm.nih.gov/nuccore/MN695296 |
| *Prunus jamasakura* | 23 | 18 | 2 | 3 | 46 | MN652612 | https://www.ncbi.nlm.nih.gov/nuccore/MN652612 |
| *Prunus kumanoensis* | 23 | 19 | 2 | 4 | 48 | MN245147 | https://www.ncbi.nlm.nih.gov/nuccore/MN245147 |
| *Prunus leveilleana* | 23 | 19 | 2 | 4 | 48 | MN913372 | https://www.ncbi.nlm.nih.gov/nuccore/MN913372 |
| *Prunus matuurae* | 23 | 20 | 1 | 7 | 51 | NC045230 | https://www.ncbi.nlm.nih.gov/nuccore/NC_045230 |
| *Prunus maximowiczii* | 24 | 20 | 2 | 3 | 49 | NC026981 | https://www.ncbi.nlm.nih.gov/nuccore/NC_026981 |
| *Prunus pensylvanica* | 23 | 19 | 1 | 5 | 48 | MN427872 | https://www.ncbi.nlm.nih.gov/nuccore/MN427872 |
| *Prunus pseudocerasus* | 22 | 16 | 1 | 3 | 42 | KX255667 | https://www.ncbi.nlm.nih.gov/nuccore/KX255667 |
| *Prunus rufa* | 26 | 16 | 0 | 2 | 44 | NC048528 | https://www.ncbi.nlm.nih.gov/nuccore/NC_048528 |
| *Prunus serrulata* var. *spontanea* | 23 | 19 | 2 | 4 | 48 | KP760073 | https://www.ncbi.nlm.nih.gov/nuccore/KP760073 |
| *Prunus speciosa* | 23 | 19 | 2 | 3 | 47 | MH998233 | https://www.ncbi.nlm.nih.gov/nuccore/MH998233 |
| *Prunus subhirtella* var. *subhirtella* | 24 | 18 | 2 | 3 | 47 | KP760075 | https://www.ncbi.nlm.nih.gov/nuccore/KP760075 |
| *Prunus takesimensis* | 23 | 18 | 1 | 5 | 47 | NC039379 | https://www.ncbi.nlm.nih.gov/nuccore/NC_039379 |
| *Prunus yedoensis* | 22 | 17 | 1 | 3 | 43 | NC026980 | https://www.ncbi.nlm.nih.gov/nuccore/NC_026980 |
