# Supplementary material for: Comparative analysis of the complete plastid genomes in Prunus subgenus Cerasus (Rosaceae): Molecular structures and phylogenetic relationships
Source: PLoS One. 2022 Apr 6;17(4):e0266535. doi: 10.1371/journal.pone.0266535 (PMC8985974; doi:10.1371/journal.pone.0266535)
Supplement: S5 Table — (DOCX) [file pone.0266535.s008.docx]

**S5 Table.** Summary for all the information of 33 outgroup species

| **Species** | **NCBI accession number** | **URL** | **Size** | **Source** |
| --- | --- | --- | --- | --- |
| *Adenostoma fasciculatum* | KY387915 | https://www.ncbi.nlm.nih.gov/nuccore/KY387915 | 133243 | New Phytologist (2017) |
| *Agrimonia pilosa* | MT040192 | https://www.ncbi.nlm.nih.gov/nuccore/MT040192 | 155188 | Mitochondrial DNA B Resour (2020) |
| *Aruncus dioicus* | KY419926 | https://www.ncbi.nlm.nih.gov/nuccore/KY419926 | 131640 | New Phytologist (2017) |
| *Chamaebatiaria millefolium* | KY420017 | https://www.ncbi.nlm.nih.gov/nuccore/KY420017 | 133124 | New Phytologist (2017) |
| *Cotoneaster horizontalis* | MN577893 | https://www.ncbi.nlm.nih.gov/nuccore/MN577893 | 159548 | Frontiers in Plant Science (2020) |
| *Crataegus kansuensis* | MF784433 | https://www.ncbi.nlm.nih.gov/nuccore/MF784433 | 159865 | Molecular Phylogenetics and Evolution (2020) |
| *Cydonia oblonga* | KX499857 | https://www.ncbi.nlm.nih.gov/nuccore/KX499857 | 159609 | New Phytologist (2017) |
| *Dryas drummondii* | KY419952 | https://www.ncbi.nlm.nih.gov/nuccore/KY419952 | 131669 | New Phytologist (2017) |
| *Elaeagnus glabra* | MN306572 | https://www.ncbi.nlm.nih.gov/nuccore/MN306572 | 152529 | Mitochondrial DNA B Resour (2020) |
| *Gillenia stipulata* | MN068263 | https://www.ncbi.nlm.nih.gov/nuccore/MN068263 | 159575 | Molecular Phylogenetics and Evolution (2020) |
| *Holodiscus argenteus* | KY420013 | https://www.ncbi.nlm.nih.gov/nuccore/KY420013 | 131042 | New Phytologist (2017) |
| *Humulus lupulus* | KT266264 | https://www.ncbi.nlm.nih.gov/nuccore/KT266264 | 153751 | Mitochondrial DNA (2015) |
| *Kerria japonica* | MN418902 | https://www.ncbi.nlm.nih.gov/nuccore/MN418902 | 160007 | Mitochondrial DNA B Resour (2019) |
| *Lyonothamnus floribundus* | KY420005 | https://www.ncbi.nlm.nih.gov/nuccore/KY420005 | 132262 | New Phytologist (2017) |
| *Malus doumeri* | KX499861 | https://www.ncbi.nlm.nih.gov/nuccore/KX499861 | 159584 | New Phytologist (2017) |
| *Malus prattii* | NC043902 | https://www.ncbi.nlm.nih.gov/nuccore/NC_043902 | 160239 | Mitochondrial DNA B Resour (2019) |
| *Neillia gracilis* | KY420006 | https://www.ncbi.nlm.nih.gov/nuccore/KY420006 | 132928 | New Phytologist (2017) |
| *Neillia serratisepala* | KY419969 | https://www.ncbi.nlm.nih.gov/nuccore/KY419969 | 133105 | New Phytologist (2017) |
| *Potentilla chinensis* | MN871983 | https://www.ncbi.nlm.nih.gov/nuccore/MN871983 | 157117 | Mitochondrial DNA B Resour (2020) |
| *Prunus andersonii* | KY419981 | https://www.ncbi.nlm.nih.gov/nuccore/KY419981 | 131183 | New Phytologist (2017) |
| *Prunus armeniaca* | KY101151 | https://www.ncbi.nlm.nih.gov/nuccore/KY101151 | 157951 | Horticulture Research (2019) |
| *Prunus mume* | MH700953 | https://www.ncbi.nlm.nih.gov/nuccore/MH700953 | 157916 | Horticulture Research (2019) |
| *Prunus persica* | HQ336405 | https://www.ncbi.nlm.nih.gov/nuccore/HQ336405 | 157790 | Molecular Biology and Evolution (2011) |
| *Prunus salicina* | NC047442 | https://www.ncbi.nlm.nih.gov/nuccore/NC_047442 | 157916 | Horticulture Research (2019) |
| *Pyracantha fortuneana* | MK920290 | https://www.ncbi.nlm.nih.gov/nuccore/MK920290 | 160388 | Journal of Systematics and Evolution (2019) |
| *Pyrus spinosa* | HG737342 | https://www.ncbi.nlm.nih.gov/nuccore/HG737342 | 159161 | PloS One (2014) |
| *Rhodotypos scandens* | KY419951 | https://www.ncbi.nlm.nih.gov/nuccore/KY419951 | 131708 | New Phytologist (2017) |
| *Rosa rugosa* | MK641521 | https://www.ncbi.nlm.nih.gov/nuccore/MK641521 | 157110 | Mitochondrial DNA B Resour (2019) |
| *Sorbaria sorbifolia* | KY419928 | https://www.ncbi.nlm.nih.gov/nuccore/KY419928 | 134424 | New Phytologist (2017) |
| *Sorbus amabilis* | MT357029 | https://www.ncbi.nlm.nih.gov/nuccore/MT357029 | 160006 | Mitochondrial DNA B Resour (2020) |
| *Spiraea martini* | KY419987 | https://www.ncbi.nlm.nih.gov/nuccore/KY419987 | 129734 | New Phytologist (2017) |
| *Ulmus chenmoui* | MG581403 | https://www.ncbi.nlm.nih.gov/nuccore/MG581403 | 159777 | Conservation Genetics Resources (2018) |
| *Ziziphus jujuba* | KU351660 | https://www.ncbi.nlm.nih.gov/nuccore/KU351660 | 161466 | Current Genetics (2016) |
