# Supplementary material for: Comparative analysis of the complete plastid genomes in Prunus subgenus Cerasus (Rosaceae): Molecular structures and phylogenetic relationships
Source: PLoS One. 2022 Apr 6;17(4):e0266535. doi: 10.1371/journal.pone.0266535 (PMC8985974; doi:10.1371/journal.pone.0266535)
Supplement: S1 Fig — (PDF) [file pone.0266535.s001.pdf]

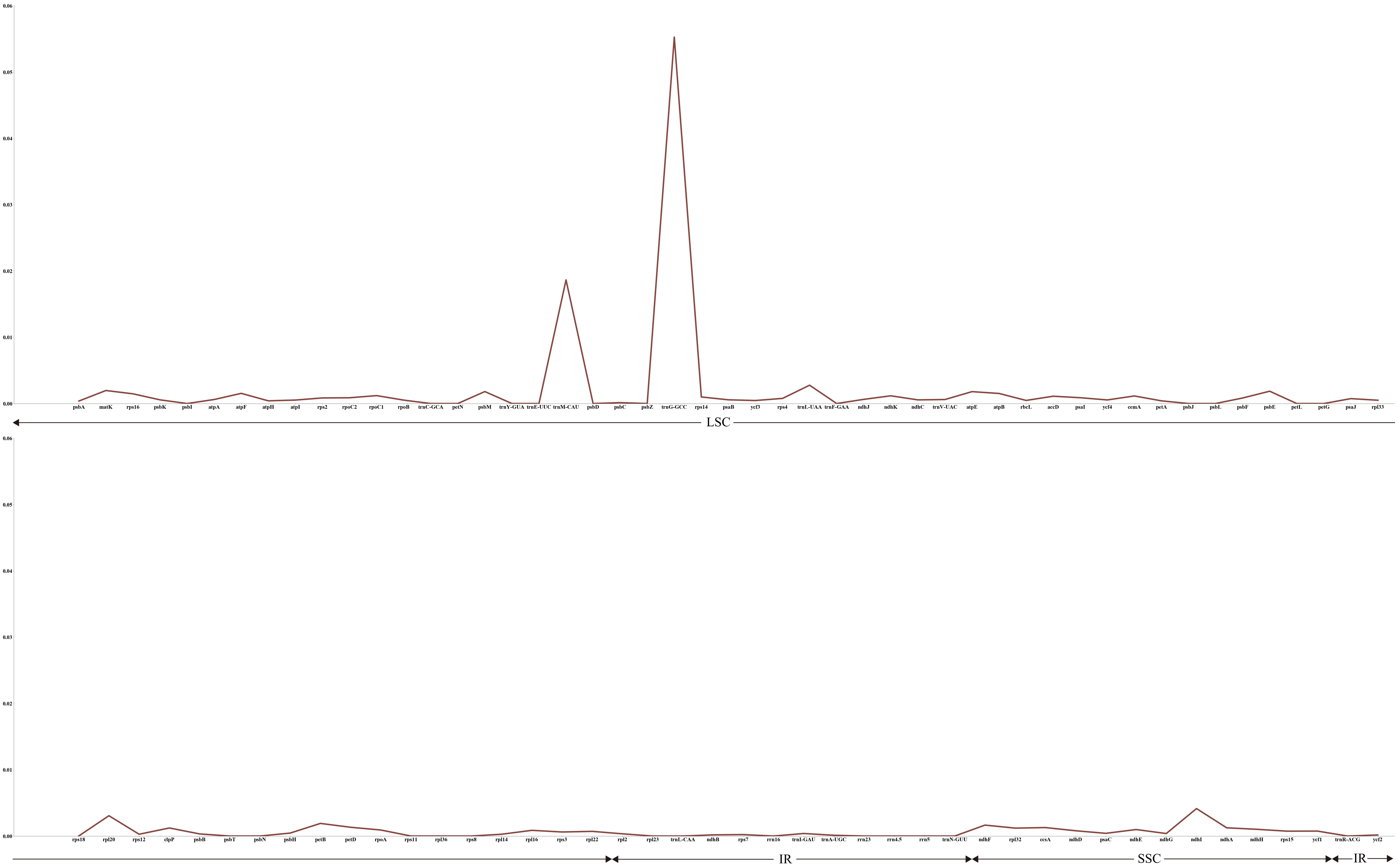
0.06
0.05
0.04
0.03
0.02
0.01
0.00
psbA
matK
rps16
psbK
psbI
atpA
atpF
atpH
atpI
rps2
rpoC2
rpoC1
rpoB
trnC-GCA
petN
psbM
trnY-GUA
trnE-UUC
trnM-CAU
psbD
psbC
psbZ
trnG-GCC
rps14
psaB
ycf3
rps4
trnL-UAA
trnF-GAA
ndhJ
ndhK
ndhC
trnV-UAC
atpE
atpB
rbcL
accD
psaI
ycf4
cemA
petA
psbJ
psbL
psbF
psbE
petL
petG
psaJ
rpl33
LSC
0.06
0.05
0.04
0.03
0.02
0.01
0.00
rps18
rpl20
rps12
clpP
psbB
psbT
psbN
psbH
petB
petD
rpoA
rps11
rpl36
rps8
rpl14
rpl16
rps3
rpl22
rpl2
rpl23
trnL-CAA
ndhB
rps7
rrn16
trnI-GAU
trnA-UGC
rrn23
rrn4.5
rrn5
trnN-GUU
ndhF
rpl32
ccsA
ndhD
psaC
ndhE
ndhG
ndhI
ndhA
ndhH
rps15
ycf1
trnR-ACG
ycf2
IR
SSC
IR
